# Viral Load as Predictor of Crimean-Congo Hemorrhagic Fever Outcome

**DOI:** 10.3201/eid1311.070222

**Published:** 2007-11

**Authors:** Darja Duh, Ana Saksida, Miroslav Petrovec, Salih Ahmeti, Iusuf Dedushaj, Marcus Panning, Christian Drosten, Tatjana Avšič-Županc

**Affiliations:** * Institute of Microbiology and Immunology, Ljubljana, Slovenia; † Clinic of Infectious Diseases, Pristina, Kosovo; ‡ National Institute of Public Health, Pristina, Kosovo; § Bernhard Nocht Institute for Tropical Medicine, Hamburg, Germany

**Keywords:** Crimean-Congo hemorrhagic fever, real-time RT-PCR, viral load, serology, dispatch

## Abstract

We used quantitative real-time reverse transcription–PCR to measure viral load in serum from 24 patients in Kosovo who had acute Crimean-Congo hemorrhagic fever. Viral load correlated with clinical disease and antibodies and could be used as a predictor of disease outcome.

Crimean-Congo hemorrhagic fever (CCHF), caused by CCHF virus, is a potentially fatal infection in Africa, Asia, Eastern Europe, and the Middle East. CCHF virus is transmitted to humans by bites of *Ixodid* ticks and from person to person by contact with blood or blood-containing body fluids. Therefore, nosocomial and intrafamiliar cases are frequently reported in CCHF outbreaks (*1*). CCHF can be treated with ribavirin, but the decision about which CCHF patients should be given the drug may be difficult (*2*,*3*). Classification of patients according to criteria of disease severity is an important step in deciding whether to initiate antiviral therapy and is based on clinical data and biochemical test results (*2*,*4*). Because of severe side effects from ribavirin treatment, early laboratory confirmation would be desirable to establish stronger criteria for case classification of CCHF.

Real-time PCR has enabled measurement of viral loads, monitoring of antiviral treatment effects and emergence of antiviral resistant strains, and prediction of disease progression and outcome (*5*). For many viral diseases, including hemorrhagic fevers, viral load measurement has become an integral part of disease management (*6*–*9*). However, to our knowledge, the usefulness of viral load monitoring for CCHF has never been investigated. Given the importance of predicting CCHF disease severity and risk for death, our aim was to measure viral load in CCHF patients and to correlate it with other laboratory parameters and disease outcomes.

## The Study

Serum samples were obtained from CCHF patients from Kosovo in 2001 (www.who.int/csr/don/2001_06_29e/en), 2003, and 2005. Clinical and biochemical data were provided by the Clinic of Infectious Diseases, Pristina, Kosovo. Presence of an acute febrile syndrome characterized by malaise, nausea, fever, and bleeding from various sites was reported as well as possible modes of infection. Leukocyte and platelet counts, aspartate and alanine aminotransferase levels, activated partial thromboplastin times, and creatinine values were available for most patients. Patients were categorized into 3 groups according to disease severity: fatal, severe, or moderate cases. On the basis of classification by Swanepoel (*4*), severe cases were defined by the presence of hemorrhagic manifestations (epistaxis, hematemesis, and melena), lowered blood pressure (<100/60 mm Hg), and raised serum creatinine and transaminase levels.

Serologic testing for anti-CCHF virus immunoglobulin (Ig) M and IgG was done by ELISA. Molecular data were obtained by real-time reverse transcription–PCR (RT-PCR) with a limit of detection of 240 copies/mL of sample as recently described (*10*). Some assay modifications were necessary for accurate quantitation of viral load. Synthetic RNA was generated as a quantitative calibrator, and a competitive internal control was constructed as previously described to detect possible influences of PCR inhibitors (*11*). The original real-time RT-PCR protocol was complemented by the addition of 200 pmol/μL of internal control probe YFP2 (5′-ROX-ATCGTTCGTTGAGCGATTAGCAG-BBQ-3′). This probe recognizes an alternative binding site introduced in the target gene by overlap-extension PCR (*11*). The standard curve for CCHF virus quantitation was based on synthetic calibrator RNA with concentrations from 24 × 10^5^ to 24 × 10^1^ copies/mL. Statistical analysis was performed with statistical software R version 2.2.1 (www.r-project.org) and the Statgraphics 5 package (Manugistics, Dresden, Germany).

A total of 24 patients had clinical, serologic, and molecular confirmation of CCHF (Table). All 24 patients had an acute febrile syndrome, 8 reported tick bites, and 2 had been exposed in a hospital. For the 9 patients who died, only 1 serum sample was available from each. For the 9 patients with severe disease and the 6 with moderate disease, 2–3 consecutive samples were available.

**Table:** Detection of CCHF viral load by real-time RT-PCR in serum of patients with acute CCHF, Kosovo*

Patient no.	Disease severity	Day of illness	IgM titer	IgG titer	Viral load (copies/mL)
1	Fatal	2	Neg	Neg	6.5100 × 10^8^
2	Fatal	3	Neg	Neg	2.5040 × 10^9^
3	Fatal	4	Neg	Neg	2.7400 × 10^9^
4	Fatal	9	Neg	Neg	3.3840 × 10^9^
5	Fatal	4	1,600	Neg	1.0160 × 10^8^
6	Fatal	6	3,200	Neg	1.3450 × 10^10^
7	Fatal	7	400	Neg	1.8675 × 10^9^
8	Fatal	7	800	Neg	3.4800 × 10^9^
9	Fatal	7	800	Neg	1.2920 × 10^9^
10	Severe	8	>6,400	Neg	1.6050 × 10^6^
		18	>6,400	400	1.1500 × 10^5^
11	Severe	9	Neg	Neg	2.3250 × 10^7^
		24	>6,400	>6,400	Neg
12	Severe	4	800	Neg	3.3900 × 10^6^
		16	6,400	3,200	Neg
13	Severe	2	Neg	Neg	1.0430 × 10^9^
		9	>6,400	400	3.1200 × 10^3^
		42	> 6,400	3,200	Neg
14	Severe	8	Neg	Neg	8.1000 × 10^6^
		14	6,400	Neg	2.0100 × 10^3^
15	Severe	3	Neg	Neg	3.8100 × 10^7^
		14	6,400	200	Neg
16	Severe	3	>1,600	Neg	2.5235 × 10^6^
		6	>800	>800	2.2350 × 10^4^
17	Severe	4	Neg	Neg	3.3600 × 10^7^
		10	>6,400	100	3.8100 × 10^4^
18	Severe	2	Neg	Neg	7.8500 × 10^7^
		9	6,400	400	3.2000 × 10^3^
		23	>6,400	>6,400	Neg
19	Moderate	12	>6,400	Neg	5.7525 × 10^4^
		17	>6,400	800	Neg
20	Moderate	9	>6,400	1,600	1.9600 × 10^3^
		11	>6,400	3,200	Neg
21	Moderate	12	>6,400	800	1.9191 × 10^5^
		13	>6,400	3,200	1.0240 × 10^4^
		32	>6,400	>6,400	Neg
22	Moderate	10	>6,400	Neg	4.6400 × 10^4^
		19	>6,400	>6,400	1.2000 × 10^3^
		26	>6,400	>6,400	Neg
23	Moderate	9	6,400	6,400	7.6800 × 10^3^
		20	>6,400	>6,400	7.5000 × 10^2^
24	Moderate	7	400	Neg	7.4400 × 10^5^
		18	>6,400	6,400	Neg

*CCHF, Crimean-Congo hemorrhagic fever: RT-PCR, reverse transcription–PCR; Ig, immunoglobulin; neg, negative result.

From the 24 patients, 43 serum samples were tested by real-time RT-PCR and ELISA (Table). Viral loads ranged from 10^2^ to 10^10^ copies per milliliter of serum, depending on the day of illness, the severity of disease, and the results of serologic analyses (Table). Whether early laboratory findings could serve as prognostic markers for outcome was explored. Because prognostic information is most relevant in the first week of disease, only samples taken up to day 7 of symptoms from any patient were included in the analysis. Average sampling days did not differ between patients who died and those who survived (5.0 and 3.9 days, respectively, p = 0.28, analysis of variance [ANOVA]). No patient from either group had detectable IgG in the first week. Although we suspected that early development of IgM might correlate with good outcome, such correlation was not found. Of those patients in whom IgM was detected up to day 7, 5 (62%) died. Of those without IgM in the first week, 3 (42%) died (insignificant difference in 1-way ANOVA, p = 0.6). Furthermore, the presence of IgM in the first week did not correlate with viral load, which suggests that virus levels in the first week can be regarded as an independent prognostic parameter. Namely, viral load seemed to be strongly related to the clinical classification (p<0.001), with the average log value 9.25 (1.78 × 10^9^) in the group of patients who died and 6.91 (8.06 × 10^6^) in the group who survived (Figure).

**Figure:**
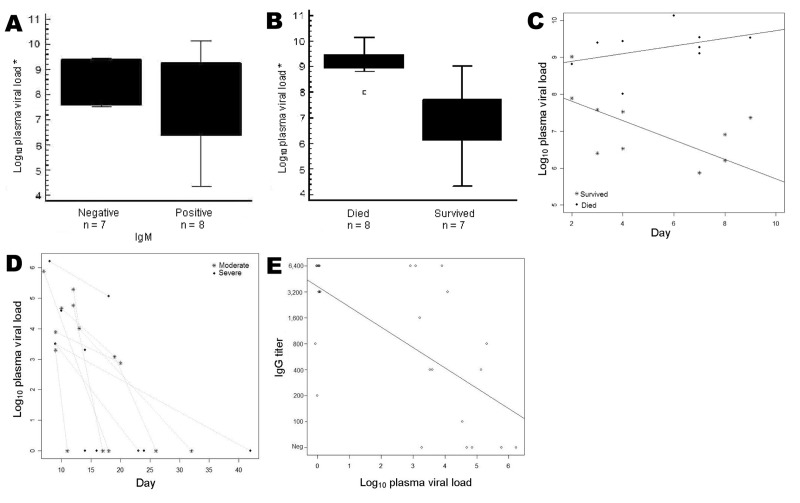
Correlation between clinical outcome, serologic data, and Crimean-Congo hemorrhagic fever (CCHF) viral load measurements. A) Viral load versus immunoglobulin (Ig) M result taken during the first week of illness. B) Viral load versus outcome. Average viral loads were 1.6 × 10^9^ copies/mL in persons who died and 5 × 10^6^ copies/mL in persons who survived (difference highly significant, p<0.0001). The dot is a datum point that has been identified as an outlier. C) Statistically significant difference (p<0.001) in CCHF viral load and day of illness between group who died and group who survived. D) No correlation in viral load and day of illness between severe and moderate CCHF cases. E) Inverse correlation of quantitative IgG levels with viral loads (p<0.0001) in samples taken after first week of illness. Black dot, >1 sample; *, first week samples.

To determine whether IgG could influence viremia, viral loads were correlated with log-transformed reciprocal antibody titers. Quantitative IgG levels showed a highly significant inverse correlation with viral loads (p<0.0001) (Figure). It was thus reasoned that IgG levels could influence the later course of disease and, in particular, could reflect a discrimination between severe and moderate cases. In samples from both categories, no relationship between IgG and clinical classification (p = 0.65) was determined after day 7. Also, no significant relationship was found between clinical classification and viral load (p = 0.74) in severe versus moderate cases. On average, viral load log value was 2.38 in severe cases and 2.69 in moderate cases (Figure).

## Conclusions

This study describes the differential influences of CCHF viral load, IgM, IgG, and clinical outcome. CCHF viral load, but not IgM, could be used as a predictor of CCHF outcome. It was unexpected that IgM correlated with neither outcome nor viral load. On the contrary, quantitative IgG levels inversely correlated with viral loads, which suggests that IgG might neutralize virus in vivo. The fact that virus titers decreased in survivors independent of antibodies during the first week implies involvement of innate or cellular immune mechanisms in the elimination of CCHF virus.

Viral load >10^8^ copies/mL is a strong factor (p<0.001) for differentiating CCHF patients who died from those who survived. However, viral load does not help differentiate between severe and moderate cases according to common case definitions (*4*). The same was true for IgM levels. Viral load is also useful for estimating need for infection control measures. Viral loads measured in our patients were high, >10^9^ copies/mL, higher than viral loads in other arboviral diseases that are not easily transmitted in the hospital, e.g., dengue (*12*). This finding could help explain why CCHF virus causes nosocomial infections on a regular basis. Another use for this finding is systematic monitoring of patients receiving ribavirin therapy. In the absence of sufficiently large numbers of treated patients, however, we could not investigate this application.
